# Quantitative Modelling of Biohydrogen Production from Indian Agricultural Residues via Dark Fermentation

**DOI:** 10.1002/open.202400095

**Published:** 2025-03-29

**Authors:** Tanmay J. Deka, Ahmed I. Osman, Mohamed Farghali, Ahmed Alengebawy, Debendra C. Baruah, David W. Rooney

**Affiliations:** ^1^ School of Chemistry and Chemical Engineering Queen's University Belfast Belfast United Kingdom; ^2^ School of Engineering, Technology Design, Canterbury Christ Church University, Canterbury Canterbury CT1 1QU UK; ^3^ Department of Agricultural Engineering and Socio-Economics Kobe University Kobe 657-8501 Japan; ^4^ Department of Animal and Poultry Hygiene & Environmental Sanitation Faculty of Veterinary Medicine Assiut University Assiut 71526 Egypt; ^5^ College of Engineering Huazhong Agricultural University 430070 Wuhan China; ^6^ Department of Energy Tezpur University 784001 Tezpur India

**Keywords:** Biohydrogen, Dark fermentation, Crop residue, Quantitative modelling, Bioenergy

## Abstract

BioH_2_, a modern biofuel with clean energy attributes and effective waste management capabilities, emerges as a promising energy source. This study employs quantitative modelling to evaluate India's bioH_2_ production potential from major crop residues. Among the seven selected crop residues, West Bengal, Uttar Pradesh, and Karnataka stand out as the top three states with surplus crop residues. The annual estimated bioH_2_ generation potential, without pretreatment, reaches approximately 103 PJ, a figure that soars to around 300 PJ with pretreatment, representing a remarkable 191 % improvement. The study underscores the effectiveness of pretreatment methods involving acid, alkali, or heat in enhancing bioH_2_ production. Despite these promising findings, efficiency‐related challenges, including temperature, pH, and pretreatment factors, are recognised. The study proposes further research and decentralised production projects as potential strategies to address these challenges, enhancing India's energy security by reducing dependence on imported fossil fuels.

## Graphical abstract

This study employs quantitative modelling to assess India's biohydrogen (BioH_2_) potential from crop residues, revealing a 191 % production boost with pretreatment, reaching ~300 PJ annually. Key surplus‐producing states are identified, highlighting BioH_2_’s role in energy security and fossil fuel reduction. Findings support sustainable, decentralised production, strengthening India's clean energy future.

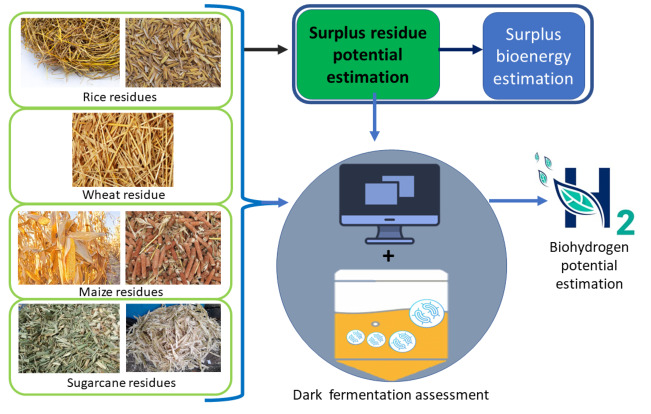

## 1 Introduction

The expanding global population in recent decades has driven greater utilisation of fossil fuels to meet escalating energy demands. However, this widespread reliance on fossil fuels across industry, electricity generation, and transportation concurrently yields greenhouse gases (GHGs), contributing to global warming and climate change.[^1^, ^2^] More than 175 nations have ratified the Paris Agreement, which aims to keep global warming to 1.5 °C over pre‐industrial levels.^[3]^ Meanwhile, it is predicted that by 2050, global energy consumption will reach 600–1000 EJ^[4]^ compared to 595 EJ in 2021, with an average increase of 25.6 %.^[5]^ Consequently, scientists are actively exploring alternative, sustainable, renewable, affordable, efficient, and low‐emission energy sources due to concerns about energy security, sustainability, and environmental protection.^[6]^ Among renewable energy sources, biomass energy has garnered significant attention as a practical means to reduce global GHG emissions.^[7]^ Although biomass currently accounts for only 10 % of the world's energy supply, its potential impact in the future is substantial, particularly in light of the growing energy demand.^[8]^ Biofuels, as a product derived from biomass, offer a compelling solution to reduce emissions when compared to the continued use of fossil‐derived fuels,[^7^, ^9^, ^10^] thus ameliorating the adverse impacts of climate change.^[11]^

Among the various biofuel options, such as biohydrogen (bioH_2_), biogas, bioethanol, and biodiesel, bioH_2_ production from biomass stands out as a highly promising method.^[12]^ Unlike first and second‐generation biofuels, bioH_2_ production from biomass does not compete with food or animal feed and does not require fertile soil.^[6]^ Agricultural residues, including rice straw, corn cobs, and wheat straw, are particularly attractive feedstocks for bioenergy production, as they are abundant and possess a 33 % bioenergy potential.[^9^, ^13^] In the context of India, a major agricultural and energy‐producing nation following China, there has been a growing shift towards renewable energy sources to meet energy needs.^[14]^ Additionally, in 2009 India adopted its first biofuel policy and acknowledged the value of biofuels.^[8]^ Nevertheless, fossil fuels still dominate India's energy portfolio, aligning with global trends. India's 57.3 % coal, 20.1 renewables, 13.2 % hydropower, 7.2 natural gas, 2 % nuclear power, and 0.2 oil.^[15]^ Despite these efforts, India's overall CO_2_ emissions totalled 2,310 million tonnes (MT), representing approximately 335.75 % of the 1990s level.^[16]^ Recent data reveals a 10 % increase in primary energy consumption and a 12 % increase in carbon emissions in 2021 compared to pre‐pandemic (COVID‐19) levels in 2019.^[17]^ During 2017 and 2018, an estimated 116 MT of crop residue was openly burned in India, resulting in the release of approximately 176.1 MT of CO_2_.^[18]^ This unregulated biomass burning contributes significantly to rising GHG emissions and exacerbates global warming,^[19]^ making India accountable for 26.2 % of global air pollution and causing approximately 1.24 million deaths within the country in 2017.^[20]^ Therefore, addressing the issue of biomass burning is crucial, as it contributes significantly to environmental pollution through the emission of harmful pollutants, including atmospheric black carbon.^[21]^ Additionally, India possesses a substantial land area of approximately 39.24 million hectares classified as wasteland, presenting a potential opportunity for the expansion of bioenergy production. Deka et al.^[22]^ conducted an assessment of India's potential for bioenergy and syngas generation using agricultural residues, drawing on production data from the 2017–18 fiscal year. Edrisi and Abhilash^[23]^ further delved into the sustainable utilisation of degraded land for bioenergy production in India, demonstrating its viability as a solution to effectively reconcile the competing demands of fuel supply and food security. This strategy garnered support due to its multifaceted benefits. Usmani^[8]^ underscored the transformative potential of these marginal lands, highlighting their capacity to yield 230–720 MT of energy crops annually, with an average annual energy potential of 6–7 EJ under similar conditions. Furthermore, the Indian Centre for High Technology (CHT) of the Ministry of Petroleum & Natural Gas (MOP&NG) allocated a substantial grant exceeding Rs. 779 million, equivalent to $9.5 million, to fund 11 hydrogen projects. These projects encompass eight completed initiatives, collectively aimed at advancing the widespread adoption of hydrogen fuel.^[24]^ Quantifying region‐wise agricultural residues for bioH_2_ production in India is critically important for promoting sustainable development and enhancing the country's energy security, while addressing pressing environmental challenges. In the coming years, investments by automakers are projected to exceed $100 million in the development of fuel‐cell automobiles.^[25]^ Overall, India has launched several initiatives to promote hydrogen production; some of these initiatives are listed in Table 1.

**Table 1:** Indian initiatives and roadmaps of hydrogen production promotion.^[24]^

Initiative year	Organisation	Description
2003	National Hydrogen Energy Board (NHEB)	The initiative addresses technological gaps in hydrogen energy generation, storage, transportation, delivery, applications, safety, codes, standards, and capacity building.
2005	Indian Oil Corporation	The inaugural hydrogen‐CNG (HCNG) fuelling station in India has been established by the Indian Oil Corporation at its Research and Development Centre located in Faridabad, close to Delhi.
2008	Tata Motors and the Indian Space Research Organisation	Tata Motors and the Indian Space Research Organisation produced a hydrogen‐powered bus, a major transportation milestone. This achievement follows years of research.
2012	Mahindra & Mahindra Ltd	The Mahindra Verito has been transformed into India's inaugural hydrogen‐powered vehicle.
2015	Air Products & Chemicals Inc.	Air Products & Chemicals Inc. has unveiled the first inaugural solar‐powered renewable fuelling station in India.
2019	SRM University and the Indigenous Coach Factory (ICF)	SRM University and ICF signed a memorandum of understanding (MoU) to create a hydrogen fuel cell train in India.
2022	Union Cabinet	The Union Cabinet approved the National Green Hydrogen Mission to make India a global leader in green hydrogen production and supply.

The production of bioH_2_ from agricultural waste offers a sustainable alternative to the predominantly non‐renewable sources used for hydrogen production, such as coal, petroleum‐derived products, and natural gas.^[26]^ BioH_2_ is derived from renewable sources, primarily biomass, through various methods, including (i) thermochemical methods like gasification, combustion, pyrolysis, and liquefaction^[27]^ and (ii) biological methods like dark fermentation (DF), photo fermentation, and bio‐photolysis.[^28^, ^29^] Thermochemical methods exhibit a notable capability to effectively manage a diverse array of feedstocks, encompassing lignocellulosic biomass, municipal solid waste, and agricultural residues.^[30]^ Nevertheless, thermochemical processes consume significant external energy inputs, posing an energy efficiency challenge.^[31]^ On the other hand, biological methods offer notable advantages due to their capacity to effectively employ diverse organic waste substrates and renewable resources as viable feedstocks.^[28]^ DF, in particular, is highly efficient in producing hydrogen from complex organic substances. Photo fermentation and bio‐photolysis use photosynthetic microorganisms and solar energy to generate hydrogen,^[32]^ making them sustainable and environmentally friendly. However, biological methods often face limitations, such as low hydrogen yield and slower reaction rates, which impact their overall efficiency.^[33]^

Among the methods mentioned, DF stands out as a promising approach due to its non‐polluting and non‐hazardous combustion characteristics.[^4^, ^29^, ^34^, ^35^] DF involves anaerobic bacteria converting carbohydrate‐rich substrates into bioH_2_ without the need for light.^[2]^ These substrates include biomass, sugar‐containing agricultural residues, wastewater, and municipal solid waste.[^2^, ^36^, ^37^, ^38^, ^39^] The process comprises several metabolic reactions, including hydrolysis, acidogenesis, acetogenesis, and methanogenesis.^[40]^ While DF for bioH_2_ production holds great promise, there are challenges to improving its efficiency.^[4]^ Factors like inoculum supply and pretreatment, substrate types, reactor designs, and hydrogen partial pressure play critical roles in the process.^[41]^ Furthermore, large‐scale bioH_2_ production has received limited attention due to issues like bioprocess instability, impure bioH_2_ production, and low conversion rates, leading to the formation of by‐products and hindering commercialization.^[42]^ In this context, efficient utilisation of available agricultural residues becomes crucial for the commercialisation of bioH_2_ production.

This study employs a novel quantitative modelling approach to evaluate the biohydrogen (bioH_2_) production potential of seven major agricultural residues across India's 36 regions. By focusing on raw and pretreated crop residues, the research highlights the novelty of achieving enhanced bioH_2_ yields through pre‐treatment methods. This study integrates region‐specific surplus residue data from 36 Indian states/regions to quantify the agri‐residue energy potential and identify the most promising residues and locations for bioH_2_ production. By comparing surplus bioenergy potential with bioH_2_ output and examining key challenges and opportunities, it offers valuable insights into scaling bioH_2_ production from crop residues to enhance energy security and promote sustainability in India.

## Methodology

### Principle of Dark Fermentation

In the process of DF, which occurs in a dark and oxygen‐free environment, carbohydrates‐rich materials undergo acidogenic fermentation to produce bio‐hydrogen along with effluent containing acetic acid, butyric acid, propionic acid, and alcohols like ethanol.[^37^, ^43^, ^44^] The microorganisms participating in dark fermentation can be categorised based on their sensitivity to oxygen and temperature requirements. Obligate microorganisms strictly rely on anaerobic environments, such as *Clostridium*, *Ethanoligenens*, and *Desulfovibrio* species.^[45]^ On the other hand, facultative microorganisms can thrive in both anaerobic and aerobic conditions. Common facultative dark fermentative microorganisms include *Bacillus* species, *Enterobacter*, *Citrobacter*, *Klebsiella*, and *Escherichia coli*, are common facultative dark fermentative microorganisms.^[46]^ The process begins with the hydrolysis of carbohydrate‐rich materials, breaking them down into sugar molecules, either through biological means or by applying pretreatment technologies.^[47]^ The resultant sugars, such as glucose molecules, undergo a series of metabolic events summarised in Table 2. Initially, glucose molecules react with nicotinamide adenine dinucleotide ion (NAD^+^) to form pyruvate (CH_3_COCOO^−^), H^+^, and nicotinamide adenine dinucleotide (NADH) as shown in Eq. (1).^[48]^

**Table 2:** The different stages of biochemical reactions involved in dark fermentation.

Dark fermentation biochemical reactions	Eq. No.	[Ref.]
Glycolysis		
C_6_H_12_O_6_+2 NAD^+^→2 CH_3_COCOO^−^+4H^+^+2NADH	1	[48]
Pyruvate: ferredoxin oxidoreductase (Pfor pathway)		
CH_3_COCOO^−^+CoA+2Fd_ox_→acetyl‐CoA+2Fd_red_+CO_2_	2	[49]
Fd_red_+2H^+^→Fd_ox_+H_2_	3	[51]
Pyruvate: formate lyase (Pfl pathway)		
CH_3_COCOO^−^+CoA‐H→acetyl‐CoA+HCOO^−^	4	[48, 49]
Formate: hydrogen lyase (fhl)		
HCOO^−^+H^+^→CO_2_+H_2_	5	[48]
Reactions of acetyl‐CoA		
Acetyl‐CoA+H_2_O→CH_3_COO^−^+H^+^+CoA‐H	6	[48]
Acetyl‐CoA+2NADH+2H^+^→CH_3_CH_2_OH+CoA‐H+2NAD^+^	7	[48]
Additional H_2_ formation		
NADH+H^+^→NAD^−^+H_2_	8	[53]

Pyruvate, the primary intermediate product of the DF process, is then anaerobically oxidised to acetyl coenzyme A (acetyl‐CoA) through one of two pathways,^[49]^ depending on the bacterial culture used:^[50]^ the pyruvate: ferredoxin oxidoreductase (Pfor) pathway or the pyruvate: formate lyase (Pfl) pathway, leading to hydrogen production: hydrogen lyase pathway or proton reduction. In the Pfor pathway, pyruvate is oxidised by CoA and ferredoxin oxidase (Fd_ox_) to acetyl‐CoA, ferredoxin reductase (Fd_red_), and CO_2_ (Eq. (2)).^[49]^ The Fd_red_ then oxidises to Fd_ox_ by reducing H^+^ ions to H_2_ (Eq. (3)).^[51]^ The breakdown of pyruvate by coenzyme A (CoA‐H) in the Pfl pathway results in acetyl‐CoA and formate ion (HCOO^−^)^[48]^ as shown in Eq. (4). Under acidic conditions, the formate hydrogen lyase (fhl) is activated, converting formic acid to H_2,_ as indicated in Eq. (5).[^48^, ^52^] Acetyl‐CoA produced in both the Pfl and Pfor routes leads to the synthesis of end‐products such as acetate and ethanol (Eq. (6) and Eq. (7)).^[48]^

As shown in Eq. (8), any remaining NADH generated as a by‐product during the conversion of glucose to pyruvate can be re‐oxidised to yield H_2_. However, this reaction relies on the end product: if the end product is either butyric acid or ethanol, no leftover NADH will be available for conversion to H_2_.^[51]^ As a result, with acetic acid as the end‐product, a maximum of 4 mol H_2_ may be created per mole glucose, whereas with butyric acid, only 2 mol H_2_ can be produced.[^43^, ^54^, ^55^] In practical scenarios where acetate and butyrate are present as end‐products, the yield tends to be lower than the theoretical maximum of 4 moles/mol glucose.^[51]^ In addition, as discussed below, other factors, such as inhibitors in dark fermentation, can negatively impact bioH_2_ generation.

### Selection Study Area and Feedstocks

India, the world's second‐most populous nation and the seventh largest in terms of land area, serves as the comprehensive study area for this research. In the context of the geographic scenario prevailing during 2017–18 in India, all 36 regions, comprising 28 states and 8 union territories (UTs), have been included in this study.^[56]^ Regarding the feedstocks for dark fermentations, 7 crop residues from 4 major crops cultivated in India were selected for analysis. Table 3 lists the heating values (HV), residue‐production ratios (RPR), and surplus residue fractions (SRF) for various types of crop residue from each crop. The feedstock characterisation process includes determining chemical composition, moisture content and heating value prior to experimentation. Standard techniques such as proximate and ultimate analysis are used to determine the carbon, hydrogen, nitrogen, and sulfur content of the residues in different literatures associated with this study.

**Table 3:** The 7 selected residues and their respective residue properties.

Crop name	Crop residue	Heating value (in MJ/kg)	Residue production ratio (RPR)	Surplus residue factor (SRF)	[Ref]
Rice	Rice straw	14.4	1.5	0.24	[57]
	Rice husk	15.45	0.2	0.71	[58]
Wheat	Wheat straw	17.25	1.5	0.17	[58]
Maize	Maize stalk	17.08	2	0.20	[58]
	Maize cob	18.36	0.3	0.38	[58]
Sugarcane	Sugarcane tops	14.73	0.05	0.19	[59]
	Sugarcane bagasse	17.54	0.33	0.50	[58]

### Estimation of DF Bio‐Hydrogen Production Potential

To estimate the potential for DF bioH_2_ production in this study, calculations are conducted on a regional (state/UT) basis, relying on available crop production data obtained from reports published by the Ministry of Agriculture (MoA), Government of India, for the fiscal year 2018–19.^[60]^ The total amount of residue generated during agricultural harvesting or processing phases can be referred to as the gross crop residue, whereas the residue that remains after fulfilling traditional purposes, such as for cattle bedding, cattle feed, organic fertiliser, cooking, and heating fuel, is termed as surplus crop residue. Surplus bioenergy can be defined as the amount of energy associated with the surplus residue of a crop in its biomass form. The gross crop residue of a specific crop is estimated by multiplying the crop production data (P) by the corresponding crop RPR value. Subsequently, the surplus crop residue is estimated by multiplying gross crop residue data by respective SRF values. The surplus bioenergy is estimated by multiplying surplus crop residue data by the heating value of the crop residue biomass.^[22]^ BioH_2_ energy can be defined as the amount of energy associated with the produced bioH_2_, and it is estimated by multiplying the bioH_2_ production data with the heating value of hydrogen.

The gross residue, surplus residue and surplus bioenergy are estimated using Eqs. 9–11 as follows:

(9) GR(i,j)=P(i,j)×RPR(i)

(10) SR(i,j)=GR(i,j)×SRF(i)

(11) E(i,j)=SR(i,j)×HV(i)

where, **GR (i,j), SR (i,j) and E(i,j)** are the gross residue potential, surplus residue potential, and surplus bioenergy for **i** crop residue in **j^th^** state/UT, respectively.

The DF bioH_2_ production potential and associated bioH_2_ energy potential from surplus crop residues are estimated using Eq. (12) and Eq. 13.
(12)

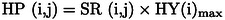

(13) HE(i,j)=HP(i,j)×HV(hydrogen)

where, **HP (i,j) and HE (i,j)** are the DF bioH_2_ production potential and DF bioH_2_ energy potential for **i** crop residue in **j^th^** state/UT, respectively. **HY(i)_max_** is the highest HY value for **i** crop residue. The hydrogen heating value (HV) considered in this study is 141.8 MJ/kg.^[61]^

Numerous researchers have studied DF bioH_2_ production using various crop residues. Table 4 shows a literature‐based summary and recalculated data of DF bioH_2_ yield (HY) through different pathways from the specific 7 crop residues considered in this study. To estimate the hydrogen production potential, the surplus residue amount for each crop residue is multiplied by their respective highest DF bioH_2_ yield value analysed from the literature. The selection of the highest DF biohydrogen (bioH_2_) yield value was undertaken to provide an overarching perspective on the maximal bioH_2_ production potential, aligning with the primary objectives of this investigation. Furthermore, the discussion section comprehensively addresses the pretreatment procedures associated with the peak DF bioH_2_ yields.

**Table 4:** DF bioH_2_ yield analysis through different pathways from the 7 crop residues considered in this study.

Substrate (Agri‐residue biomass)	Substrate pretreatment	Inoculum	Inoculum pretreatment	Operating conditions	BioH_2_ yield (HY.) (in mL/g dry biomass)	[Ref.]
Rice straw	Alkali pretreatment (Heating at 121 °C with 10 % NH_4_OH solution for 1 hr) Or Acid pretreatment (Heating at 121 °C with 1.0 % H_2_SO_4_ for 50 mins)	*Thermotoga neapolitana*	– NA –	pH=7.5 Temperature=75 °C	*Y_R_=50.85 mL/g *Y_P_=60.48 mL/g	[62, 63]
	Microwave/alkali pretreatment (Microwave heating at 140 °C with 0.5 % NaOH for 15 mins)+enzymatic hydrolysis	Anaerobic activated sludge	Boiling for 30 mins	pH=6.5 Temperature=35 °C Biomass concentration=60 g/l	*Y_P_=102.3 mL/g	[63, 64]
	Hydrothermal pretreatment (at 210 °C, 0 min holding time)	Anaerobic digested sludge	– NA –	pH=7.0 Temperature=35 °C HRT=3 days	*Y_R_=0.198 mL/g *Y_P_=18.48 mL/g	[63, 65]
Rice husk	Acid pretreatment (Heating at 121 °C with 5 % w/v H_2_SO_4_ for 1 hr)	Anaerobic granular sludge	Heating at 90 °C for 30 mins	pH=7.0–7.5 Temperature=35 °C	Y_P_=320.6 mL/g (Without enzymatic saccharification) Y_P_=473.1 mL/g (With enzymatic saccharification: 0.75 mg cellulose/mL)	[66]
Wheat straw	Ozone pretreatment for 45 mins	Cow manure & sediment slurry	Heating at 90 °C for 20 mins	pH=6.0 Temperature=35 °C	*Y_R_=32.76 mL/g *Y_P_=84.41 mL/g	[67, 68]
	Heat/acid pretreatment (Heating at 120 °C with 2 % w/v H_2_SO_4_ for 1.5 hr)	H_2_‐producing microflora	– NA –	Temperature=35 °C	*Y_R_=4.68 mL/g *Y_P_=30.56 mL/g	[68, 69]
	Heat/acid pretreatment (Microwave heating with 2 % HCl for 8 min)	Cow dung compost	Infrared oven heating for 2 hrs	Temperature=36 °C pH=6.5 Substrate concentration=25 g/L	*Y_P_=56.073 mL/g	[68, 70]
Maize stalk	Acid pretreatment (Heating at 121 °C with 0.5 wt % H_2_SO_4_ for 1 hr)	Cow dung compost	Microwave (2450 W) heating For 1.5 mins	Temperature=36 °C pH=7.0	Y_P_=129.8 mL/g	[71]
	Microwave/alkali pretreatment (Microwave heating at 2 kW with 0.12 NaOH/g biomass for 45 min)	*Clostridium thermocellum* DSM 7072 and *Clostridium thermosaccharolyticum* DSM 869	– NA –	Temperature=55 °C Liquid: solid ratio=50 : 1	Y_R_=68.2 mL/g Y_P_=105.6 mL/g	[72]
	Steam explosion at 1.6 MPa for 5 min	Anaerobic sludge	Boiled for 15 mins and augmented with *Clostridium* *Paraputrificum*	Temperature=37 °C pH=6.5	Y_R_=63.7 mL/g	[73]
	Acid pretreatment (Heating at 121 °C with 0.5 wt % H_2_SO_4_ for 1 hr)	Cow dung compost	Microwave heating (2450 W) for 1.5 min	Temperature=36 °C pH=7.0	Y_P_=144.3 mL/g	[74]
	– NA –	Cow dung compost	Microwave heating (2450 W) for 1.5 min	Temperature=36 °C Biomass=20 g/L NH_4_HCO_3_=1.76 g/L KH_2_PO_4_=0.91 g/L	Y_R_=92.9 mL/g	[75]
	Heating 108 °C with 0.75 % NaOH, for 30 mins+enzymatic hydrolysis (Cellulose: 12 IU/g biomass and hemicellulase 2400 IU/g biomass)	Cow dung	Heat shocked	Temperature=35 °C pH=7.0	Y_P_=163.1 mL/g	[76]
Maize cob	Acid/heat (1.5 % HCl, 30 min)+enzymatic hydrolysis: 6.5 U/g cellulase	Dairy manure	Infrared oven heating at 112 °C for 5 h	Temperature=6 °C pH=7.0	Y_R_=49.4 mL/g Y_P_=120.5 mL/g	[77]
	–	Municipal sewage sludge	(100 °C, 10 min)	Temperature=40 °C pH=5.5 Fermentation time=60 h	*Y_R_=93.10 mL/g (With simultaneous saccharification and fermentation)	[78, 79]
Sugarcane tops	Fungal pretreatment (White rot fungus *Pleurotus pulmonarium* MTCC 1805)	Cow dung	(100 °C, 20 min)	pH=6.5 Temperature=37 °C	*Y_R_=43.17 mL/g *Y_P_=60.60 mL/g	[80, 81]
Sugarcane bagasse	0.25 % (w/w) NaOH, 50 °C, 30 min+Enzymatic hydrolysis	Cow dung	(100 °C, 20 min)	pH=6.5 Temperature=37 °C	Y_R_=55 mL/g Y_P_=93.4 mL/g	[82]
	0.5 % HCl	*Bacillus subtilis* AuChE413		Temperature=37 °C pH=7.0 Biomass loading=1 % (w/v)	Y_P_=39.6 mL/g	[83]
	1 g/l nanoTiO_2_ (120 min UV. irradiation), 30 min 2 % (v/v) H_2_SO_4_	Anaerobic sludge	(95 °C, 15 min)	Temperature=37 °C pH=5.0	*Y_P_=99.16 mL/g	[84, 85]
	3 % NaOH (80 °C, 3 hr)	*Clostridium thermocellum* ATCC 27405		Temperature=55 °C 20 mM CaCO_3_	*Y_R_=48.63 mL/g *Y_P_=103.72 mL/g	[85, 86]

* Calculated data based on different literature, (Y_R_=bioH_2_ yield for raw substrate, Y_P_=bioH_2_ yield for pretreated substrate), HRT=Hydraulic retention time

## 2 Results and Discussion

### 2.1 Crop‐Wise Analysis of DF bioH_2_ Production Potential

#### 2.1.1 Rice

Rice is one of the most widely consumed grains in the food industry and serves as a staple food in many South Asian countries. India, being the world's second‐largest producer of rice, has achieved an estimated rice production of approximately 127 MT in the fiscal year 2022.^[87]^ Rice straw and rice husk are two key agri‐residues associated with rice production. The rice production data for 12 major states in 2018–19 has been sourced from the literature.^[60]^ The respective gross and surplus residue for rice straw and rice husk were calculated using Eq. (9) and Eq. (10). Concerning the surplus rice straw and surplus rice husk residue potential, West Bengal with 5.778 MT (rice straw) and 2.279 MT (rice husk) is at the top, followed by Uttar Pradesh (5.594 MT rice straw, 2.207 MT rice husk), Punjab (4.6152 MT rice straw, 1.82 MT rice husk), Andhra Pradesh (2.97 MT rice straw, 1.17 MT rice husk), Odisha (2.632 MT rice straw, 1.038 MT rice husk), and Telangana (2.412 MT rice straw, 0.951 MT rice husk). Table S1 shows the overall estimated data for rice crop production, gross and surplus residue estimation for rice residues, and associated bioH_2_ production and bioH_2_ energy potential.

**Figure 1:**
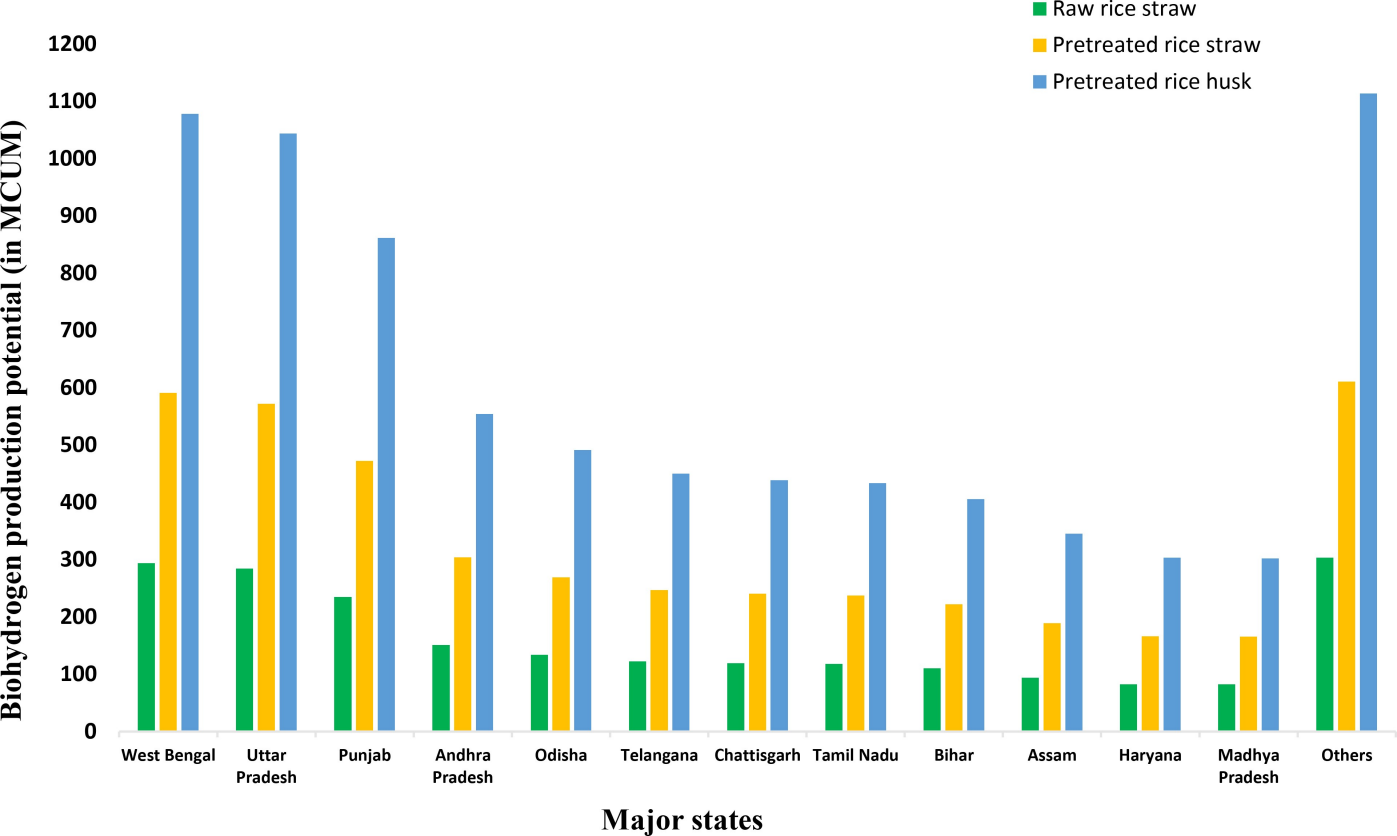
State‐wise bioH_2_ production potential from rice residues (rice straw & rice husk).

The bioH_2_ production potential data for each crop residue has been calculated using bioH_2_ yield and the surplus residue amount of the respective crop residue. The highest bioH_2_ yields from rice straw, Y_R_ and Y_P_ considered in this estimation are 50.85 and 102.3 mL/g dry biomass, respectively. The dark fermentation operating conditions for raw rice straw are a pH of 7.5 and a temperature of 75 °C. However, for pretreated rice straw, the conditions are slightly different, with a pH of 6.5 and a temperature of 35 °C. The pretreatment methods for preparing rice straw for dark fermentation include microwave/alkali pretreatment (microwave heating at 140 °C with 0.5 % NaOH for 15 mins) and enzymatic hydrolysis.^[64]^ The operating conditions for the pretreated rice husk dark fermentation include a pH of 7.0–7.5 and a temperature of 35 °C. The bioH_2_ yield for pretreated rice husk (Y_P_) is 473.1 mL/g dry biomass with enzymatic saccharification of 0.75 mg cellulose/mL. Heating at 121 °C with a 5 % w/v H_2_SO_4_ solution for 1 hour is implemented for rice husk pretreatment.^[66]^ Figure 1 shows the state‐wise bioH_2_ production potential from rice straw (raw and pretreated) and rice husk (pretreated). A trend similar to surplus residue data can be seen for the bioH_2_ production potential from rice residues, with West Bengal producing the highest amount, followed by Uttar Pradesh, Punjab, Andhra Pradesh, Odisha, Telangana, etc. The maximum bioH_2_ production potential for West Bengal is estimated as 1669.332 MCUM (considering combined data for pretreated rice straw and pretreated rice husk). The maximum bioH_2_ energy potential associated with the pretreated rice residues in West Bengal is 21.28 PJ, which is almost 13.786 % of the total estimated maximum bioH_2_ energy potential from pretreated rice residues in India. The 12 major states mentioned in Figure 1 have a maximum rice residue‐based bioH_2_ energy potential of around 132.375 PJ.

#### 2.1.2 Wheat

Wheat is another key grain in Indian agriculture, with a production of 111.32 MT recorded in the fiscal year 2022.^[87]^ Wheat straw is one of the main residues associated with wheat production, studied here.

The top 7 major states with high amounts of surplus wheat straw production in the year 2018–19 are Uttar Pradesh (8.351 MT), Punjab (4.651 MT), Madhya Pradesh (3.945 MT), Haryana (3.205 MT), Rajasthan (2.675 MT), Bihar (1.568 MT) and Gujrat (0.612 MT). Table S2 shows the overall estimated data for wheat crop production, gross and surplus residue estimation for wheat residues, and associated bioH_2_ production and bioH_2_ energy potential.

**Figure 2:**
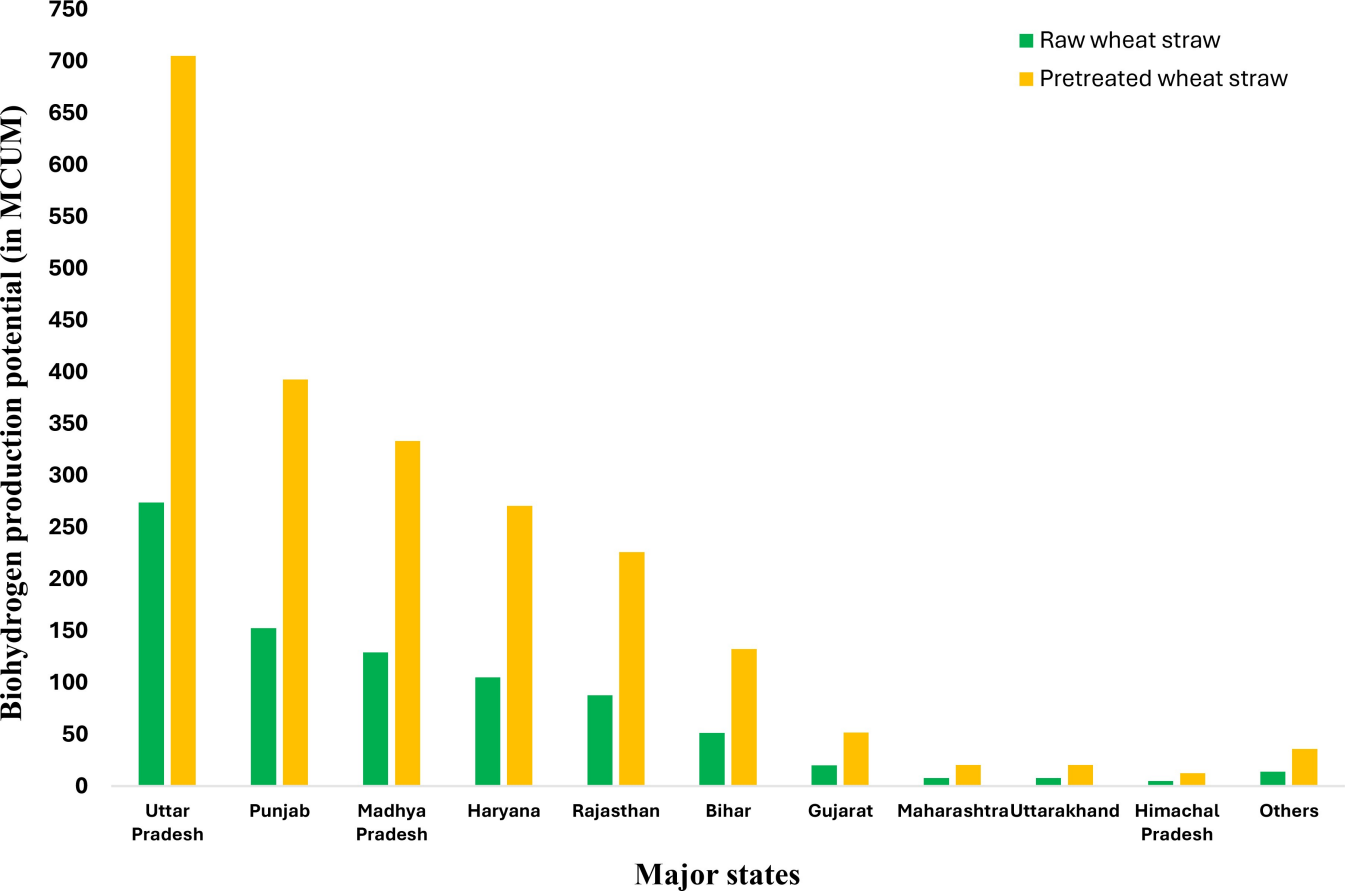
State‐wise bioH_2_ production potential from wheat straw residue.

The state‐wise bioH_2_ production potential from a wheat straw also follows a similar trend to the surplus residue trend. The operating conditions for raw and pretreated wheat straw dark fermentation include a pH of 6.0 and a temperature of 35 °C. The wheat straw bioH_2_ yields considered in this estimation are 32.76 mL/g dry biomass for Y_R_ and 84.41 mL/g dry biomass for Y_P_. Based on the yield data considered, the pretreatment approach for wheat straw involves ozone pretreatment lasting 45 minutes.[^67^, ^68^] Figure 2 shows the major states’ respective bioH_2_ production potential from raw and pretreated wheat straws. Uttar Pradesh is at the top alone, can produce an estimated maximum of 704.929 MCUM bioH_2_ (with pretreated wheat straw), equivalent to around 8.986 PJ of bioH_2_ energy, which is 32.05 % of total Indian wheat straw bioH_2_ energy potential. The top 7 major states have a maximum potential of 2110.913 MCUM of bioH_2_ (with pretreated wheat straw), equivalent to around 26.91 PJ of bioH_2_ energy, which is 95.96 % of the total Indian wheat straw bioH_2_ energy potential.

#### 2.1.3 Maize

Maize is another major crop grown in India, in the 4^th^ place on the food grain production list with an annual production of 32.42 MT recorded in the fiscal year 2022.^[87]^ Maize stalk and maize cob are two major residues associated with maize production.

In terms of surplus maize residues in 2018–2019, Karnataka is at the top with almost 1.492 MT maize stalk and 0.425 MT maize cob, which is followed by Madhya Pradesh (1.472 MT maize stalk, 0.419 MT maize cob), Bihar (1.208 MT maize stalk, 0.344 MT maize cob), Tamil Nadu (1.004 MT maize stalk, 0.286 MT maize cob), Telangana (0.812 MT maize stalk, 0.231 MT maize cob), Rajasthan (0.784 MT maize stalk, 0.223 MT maize cob), Maharashtra (0.772 MT maize stalk, 0.22 MT maize cob), etc. Table S3 shows the overall estimated data for maize crop production, gross and surplus residue estimation for maize residues, and associated bioH_2_ production and bioH_2_ energy potential.

The surplus maize stalk and cob residue directly impact the potential bioH_2_ production from the maize residues.

The bioH_2_ yields for maize stalk, represented as Y_R_ and Y_P_, are 92.9 mL/g dry biomass and 163.1 mL/g dry biomass, respectively. The operating conditions for dark fermentation of raw maize straw include a temperature of 36 °C, a biomass concentration of 20 g/L, and a pH of 7.0. For pretreated maize stalk, the conditions were almost similar, with a pH of 7.0 and a temperature of 35 °C. Again, for maize cob in this study, the corresponding Y_R_ and Y_P_ stand at 93.1 mL/g dry biomass and 120.5 mL/g dry biomass, respectively. Dark fermentation of raw maize cob required operating conditions with a pH of 5.5, a temperature of 40 °C, and a fermentation time of 60 hours. The operating conditions for pretreated maize cob dark fermentation include a pH of 7.0 and a lower temperature setting of 6 °C.

**Figure 3:**
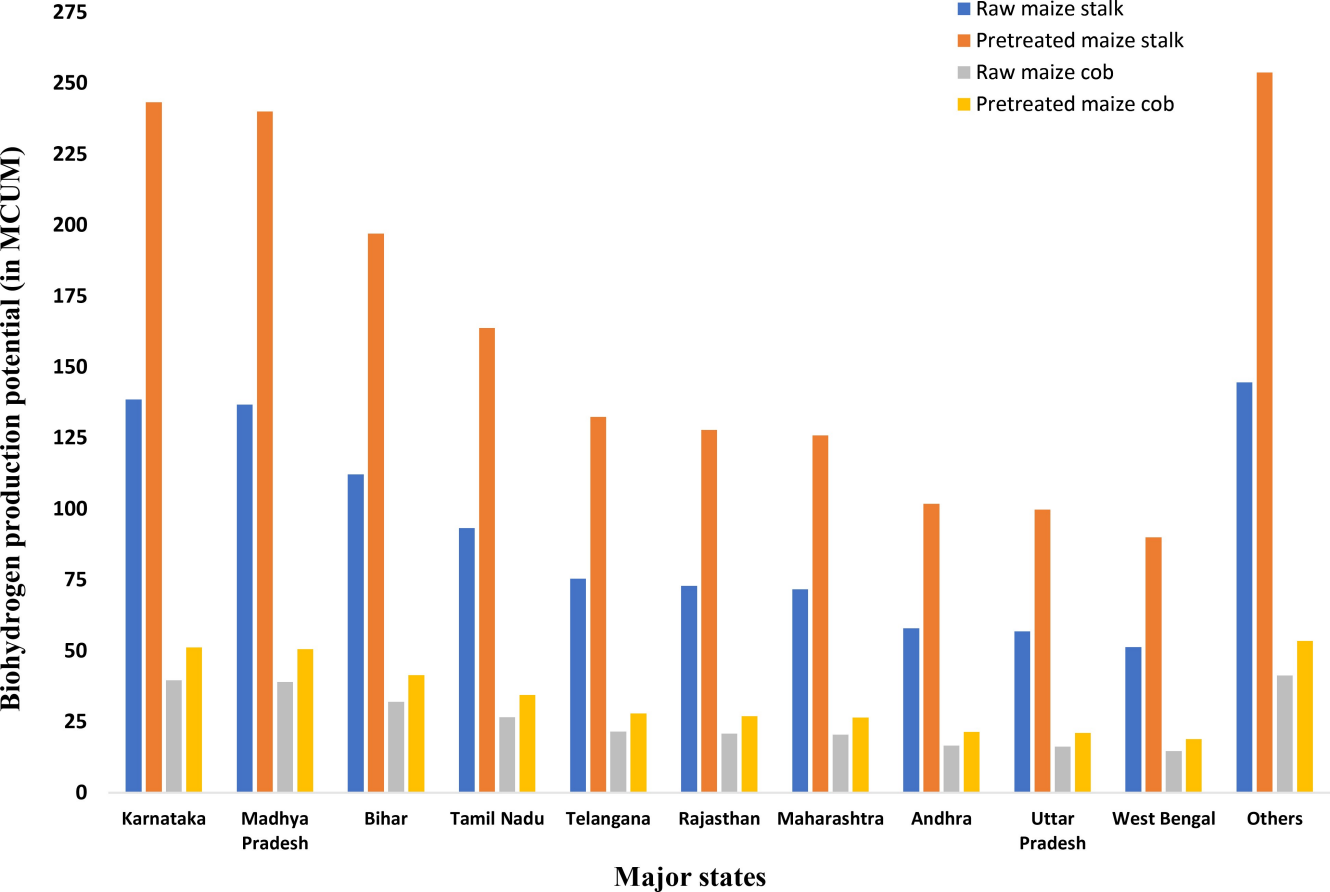
State‐wise bioH_2_ production potential from maize residues (maize stalk (raw & pretreated) and maize cob (raw & pretreated)).

The pretreatment for maize stalk involves heating at 108 °C with a 0.75 % NaOH solution for half an hour, followed by enzymatic hydrolysis utilizing cellulase (12 IU/g biomass) and hemicellulase (2400 IU/g biomass). Conversely, the maize cob undergoes a combined acid/heat treatment (1.5 % HCl, 30 minutes) before enzymatic hydrolysis, employing a cellulase concentration of 6.5 U/g.[^76^, ^77^] Figure 3 shows the state‐wise DF bioH_2_ production potential for the major maize‐producing states. It shows a similar trend to the surplus maize crop residues, and Karnataka tops the list with a maximum bioH_2_ production potential of 294.584 MCUM (with pretreated maize stalk and maize cob), equivalent to 3.755 PJ of maximum bioH_2_ energy production potential. This is followed by Madhya Pradesh (3.705 PJ), Bihar (3.041 PJ), Tamil Nadu (2.527 PJ), Telangana (2.043 PJ), Rajasthan (1.973 PJ), Maharashtra (1.943 PJ). The top 10 states shown in Figure 3 have a maximum cumulative potential of 23.498 PJ of bioH_2_ energy, which is 85.715 % of the total maximum maize residue bioH_2_ energy potential (with pretreated maize stalk & maize cob).

#### 2.1.4 Sugarcane

Another major crop in Indian agriculture is sugarcane. Sugarcane tops and bagasse are two abundant byproducts generated in various Indian states. In terms of the leading producers of excess sugarcane residues, Uttar Pradesh stands out with 1.707 MT of sugarcane tops and 29.652 MT of sugarcane bagasse. Following closely is Maharashtra, with 0.878 MT of sugarcane tops and 15.253 MT of sugarcane bagasse. Karnataka contributes 0.399 MT of sugarcane tops and 6.931 MT of sugarcane bagasse, while Tamil Nadu produces 0.154 MT of sugarcane tops and 2.675 MT of sugarcane bagasse.

The production of DF bioH_2_ from both raw and pretreated sugarcane tops, as well as from raw sugarcane bagasse, is optimised at a pH of 6.5 and a temperature of 37 °C. In contrast, the production of DF bioH_2_ from pretreated sugarcane bagasse necessitates a temperature of 55 °C and the addition of 20 mM CaCO_3_.

The considered bioH_2_ yields, Y_R_ and Y_P_, for sugarcane tops are 43.17 mL/g dry biomass and 60.6 mL/g dry biomass, respectively.[^80^, ^81^] Similarly, the Y_R_ and Y_P_ for sugarcane bagasse in this study are 55 mL/g dry biomass and 103.72 mL/g dry biomass, respectively.[^85^, ^86^] Fungal pretreatment was considered for sugarcane tops, whereas heating with 3 % NaOH solution at 80 °C for 3 h was considered for sugarcane bagasse for pretreatment.

**Figure 4:**
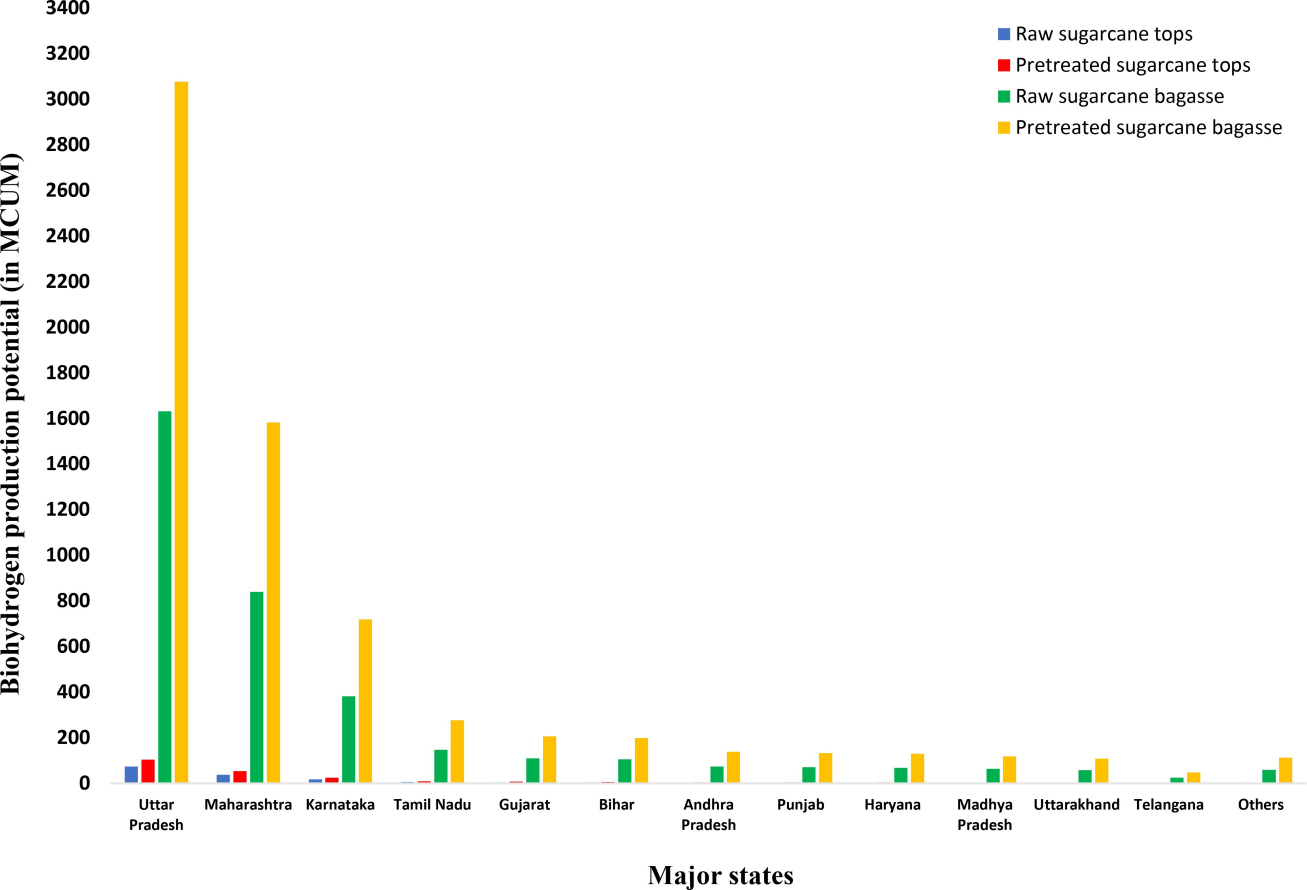
State‐wise bioH_2_ production potential from sugarcane residues (sugarcane top (raw & pretreated) and sugarcane bagasse (raw & pretreated)).

Figure 4 shows the top 12 major states with high sugarcane residue‐based bioH_2_ production potential. In terms of DF bioH_2_ production potential from sugarcane residues, Uttar Pradesh emerges as the frontrunner, capable of generating a maximum of 3178.98 MCUM of bioH_2_ (utilising pretreated sugarcane tops and pretreated sugarcane bagasse). This translates to an energy potential of 40.525 PJ of bioH_2_, representing approximately 44.91 % of India's total maximum bioH_2_ energy potential derived from sugarcane residues (with pretreated sugarcane tops and pretreated sugarcane bagasse). This is followed by Maharashtra (1635.217 MCUM, 20.845 PJ), Karnataka (743.134 MCUM, 9.473 PJ), Tamil Nadu (286.747 MCUM, 3.655 PJ), etc. Notably, the leading three states, namely Uttar Pradesh, Maharashtra, and Karnataka, alone contribute a maximum bioH_2_ energy potential of 70.843 PJ, accounting for approximately 78.51 % of India's total maximum bioH_2_ energy potential from sugarcane residues (with pretreated sugarcane tops and pretreated bagasse). Table S4 shows the overall estimated data for sugarcane crop production, gross and surplus residue estimation for sugarcane residues, and associated bioH_2_ production and bioH_2_ energy potential.

### 2.2 Total DF BioH_2_ Production Potential in India

In the previous section, the variation of surplus residue potential and their respective bioH_2_ production potential across states was described. Table 5 presents the potential for DF bioH_2_ production and bioH_2_ energy, while Table S5 provides a residue‐wise potential analysis of gross residue, surplus residue, and corresponding surplus bioenergy. The estimated gross crop residue potential of India for 2018–19 from the selected 7 residues in this study is found to be 565.889 MT. Among these residues, rice straw emerges as the highest contributor with 174.63 MT, followed by wheat straw with 153.285 MT. Sugarcane bagasse ranks third with 132.053 MT, followed by maize stalk with 54.46 MT, rice husk with 23.284 MT, sugarcane tops with 20.008 MT, and maize cob with 8.169 MT.

**Table 5:** Residue‐wise analysis of DF bioH_2_ production & energy potential.*

Agri‐residue biomass (*pretreatment type*)	Inoculum *(Pretreatment)*	DF operating condition	Considered the highest bioH_2_ yield (in mL/g dry biomass)	BioH_2_ production potential (in MCUM or Mm^3^)	Total bioH_2_ energy (in PJ)
Rice straw (*raw residue*)	Thermotoga neapolitana	pH=7.5 Temperature=75 °C	50.85	2131.185	27.168
Rice straw *((Microwave heating at 140 °C with 0*.5 % *NaOH for 15 mins)+enzymatic hydrolysis)*	Anaerobic activated sludge *(30 mins boiling*)	pH=6.5 Temperature=35 °C Biomass concentration=60 g/L	102.3	4287.516	54.656
Rice husk *((Heating at* 121 °C *with* 5 % *w/v H_2_SO_4_ for 1 hr)* *+enzymatic saccharification))*	Anaerobic granular sludge *(30 mins heating at* 90 °C*)*	pH=7.0–7.5 Temperature=35 °C	473.1	7821.119	99.702
Wheat straw (*raw residue*)	Cow manure & sediment slurry *(20 mins heating at* 90 °C*)*	pH=6.0 Temperature=35 °C	32.76	853.675	10.882
Wheat straw *(Ozone pretreatment for 45 mins)*			84.41	2199.594	28.039
Maize stalk (*raw residue*)	Cow dung compost *(Microwave heating (2450 W) for 1.5 min)*	Temperature=36 °C Biomass=20 g/L NH_4_HCO_3_=1.76 g/L KH_2_PO_4_=0.91 g/L	92.9	1011.867	12.899
Maize stalk (*Heating* 108 °C *with 0*.75 % *NaOH, for 30 mins+enzymatic hydrolysis* *(Cellulose: 12 IU/g biomass and hemicellulase* *2400 IU/g biomass))*	Cow dung *(heat shocked)*	Temperature=35 °C pH=7.0	163.1	1776.485	22.646
Maize cob *(raw residue)*	Municipal sewage sludge *(heating at* 100 °C *for 10 min)*	Temperature=40 °C pH=5.5 Fermentation time=60 h	93.1	289.003	3.684
Maize cob *(Acid/heat (1*.5 % *HCl, 30 min)+enzymatic* *hydrolysis: 6.5 U/g cellulase)*	Dairy manure *(Infrared oven heating at* 112 °C *for 5 h)*	Temperature=6 °C pH=7.0	120.5	374.058	4.768
Sugarcane tops (*raw residue*)	Cow dung *(heating at* 100 °C *for 20 min)*	pH=6.5 Temperature=37 °C	43.17	164.112	2.092
Sugarcane tops *(Fungal pretreatment with* *(White rot fungus* *Pleurotus pulmonarium MTCC 1805))*			60.60	230.372	2.937
Sugarcane bagasse (*raw residue*)	Cow dung *(heating at* 100 °C *for 20 min)*	pH=6.5 Temperature=37 °C	55	3631.452	46.293
Sugarcane bagasse *(Heating at* 80 °C *for 3 hr with* 3 % *NaOH solution)*	*Clostridium thermocellum* ATCC 27405	Temperature=55 °C 20 mM CaCO_3_	103.72	6848.258	87.300
Total			– NA –	8081.293 (min) 23537.402 (max)	103.018 (min) 300.048 (max)

* More information related to residue potential and bioenergy potential is available in Supplementary Table S5.

The surplus crop residue potential for each crop residue has been calculated using the SRF values from Table 3 and Eq. (10). In total, India is estimated to have a surplus residue potential of 168.324 MT from the seven crop residues considered in this study for the year 2018–2019. Among these residues, sugarcane bagasse has the highest national surplus residue potential, amounting to 66.026 MT. It is followed by rice straw (41.911 MT), wheat straw (26.058 MT), rice husk (16.532 MT), maize stalk (10.892 MT), sugarcane tops (3.801 MT), and maize cob (3.104 MT). The available surplus bioenergy potential from these seven major crop residues in India is estimated to be 2765.57 PJ.

In the analysis of bioH_2_ production potential, it is observed that pretreated rice husk has the highest potential, approximately 7821.12 MCUM, despite having the fourth‐highest surplus residue potential. This is attributed to the high bioH_2_ yield (473.1 mL/g dry biomass) of pretreated rice husks compared to other residues. In terms of pretreated residue‐based bioH_2_ production, sugarcane bagasse follows with the second‐highest potential at 6848.26 MCUM, followed by rice straw (4287.52 MCUM), wheat straw (2199.59 MCUM), maize stalk (1776.49 MCUM), maize cob (230.372 MCUM), and sugarcane tops (230.372 MCUM). When considering DF bioH_2_ production potential from raw residues, sugarcane bagasse leads with a maximum potential of 3631.452 MCUM, followed by rice straw (2131.19 MCUM), maize stalk (1011.87 MCUM), wheat straw (853.68 MCUM), sugarcane tops (164.11 MCUM) and maize cob (289 MCUM). The main pretreatment methods considered for practical application in this study have been mentioned in Table 5 for each studied agri‐residue.

**Figure 5:**
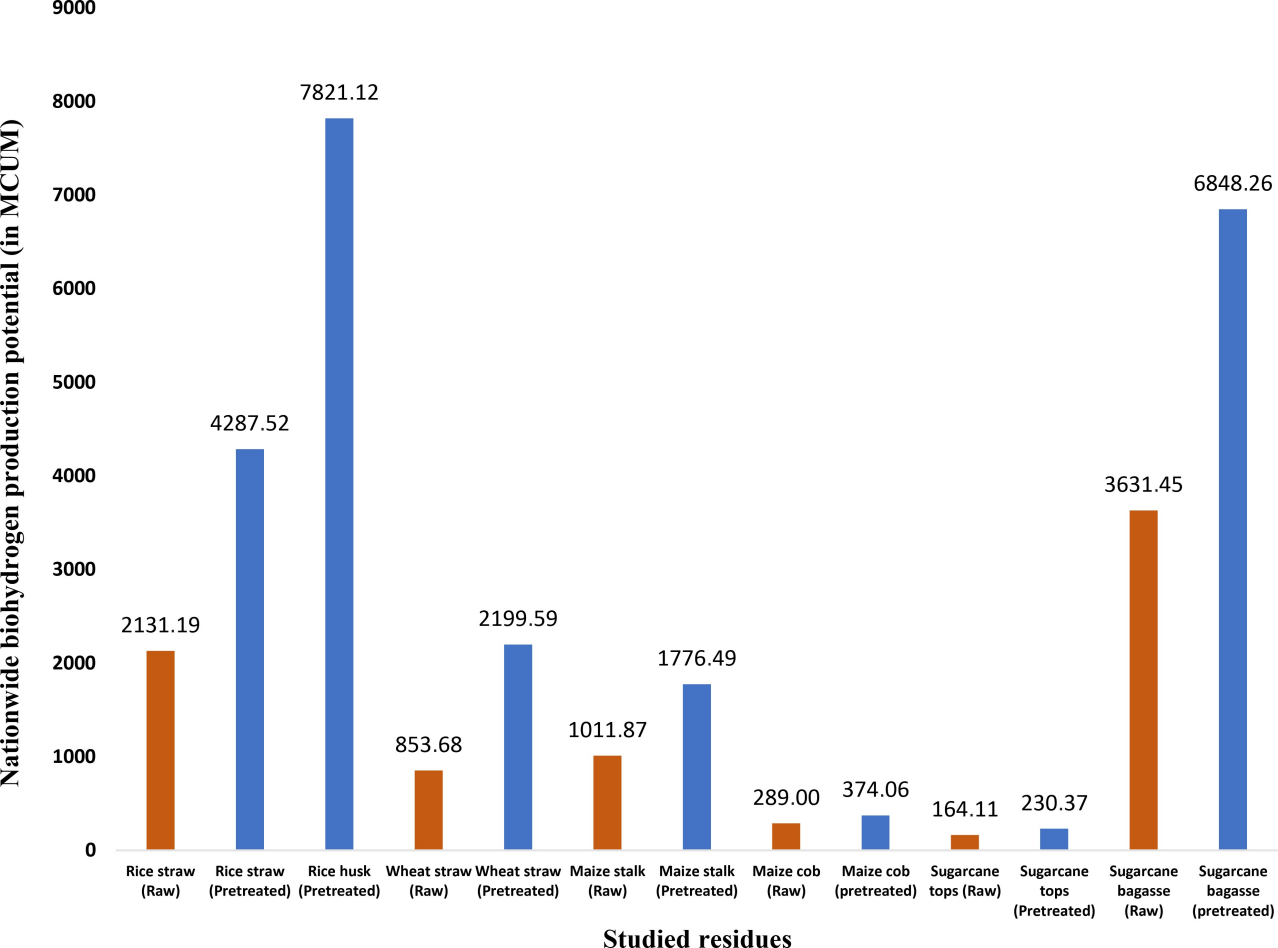
Residue‐wise DF bioH_2_ production potential in India (raw & pre‐treated).

In terms of nationwide DF bioH_2_ energy, the 6 raw crop residues discussed in this study (rice straw, wheat straw, maize stalk, maize cob, sugarcane top, and sugarcane bagasse) have a combined potential of 103.018 PJ, which can be taken as the minimum potential. However, the maximum bioH_2_ energy potential from the 7 pretreated crop residues (rice straw, rice husk, wheat straw, maize stalk, maize cob, sugarcane top, sugarcane bagasse) is estimated to be around 300.048 PJ. When compared to India's primary energy consumption data in the year 2019,^[88]^ the estimated potential bioH_2_ energy from the 7 residues could have been equivalent to 0.27–0.77 %. It is evident that pretreated rice husk and sugarcane bagasse hold the highest potential to be used as feed for bioH_2_ production in future projects. Figure 5 shows the overall nationwide DF bioH_2_ production potential from India's different studied crop residues.

## 3 Challenges and Prospects in DF BioH_2_ Production from Agricultural Residues in India

Although various agricultural residues are available in all regions of India for bioH_2_ production using DF, there are certain challenges in the whole bioH_2_ production process, as discussed in this section. Several critical process parameters influence the efficiency of bioH_2_ production using DF, including the choice of inoculum, reactor type, pH, temperature, hydraulic retention time (HRT), partial pressure of H_2_/CO_2_, substrate, and type of pretreatment.[^4^, ^89^, ^90^]

The rate of bacterial growth and metabolic activities, as well as the rate of bioH_2_ production, are directly proportional to the process temperature. DF processes generally typically perform best at temperatures ranging from 35 to 55 °C.^[4]^ For instance, Van Groenestijn et al.^[91]^ demonstrated that bioH_2_ production by bacterial species thrives under high thermophilic conditions (45–65 °C) compared to mesophilic settings (25–40 °C). However, the major challenge associated with dark fermentation is its limited yield. The highest theoretical output, under extreme thermophilic conditions at around 70 °C, is 4 mol of bioH_2_ for every mole of glucose.[^4^, ^45^, ^46^, ^92^] The production of additional end products such as propionic acid, butyric acid, acetic acid, methanol, acetone, or butanol can diminish the quantity of bioH_2_ generated.[^29^, ^92^]

Inhibitors in the DF process that impact the efficiency of bioH_2_ production can be categorised into three groups: i) biological inhibitors (e. g., thiosulfinate and bacteriocins), ii) organic inhibitors (e. g., furan, volatile fatty acids, and phenolic derivatives), and iii) inorganic inhibitors (e. g., light metals, heavy metals, sulfate, ammonia, and hydrogen gas).^[38]^ It is important to regularly remove produced bioH_2_, as its accumulation can lead to an increase in pressure, ultimately reducing the rate of its production.^[89]^

The pH level plays a significant role in bioH_2_ production through DF, with the ideal range falling between 5 and 6.^[93]^ Numerous studies have indicated that a pH of 5.5 is optimal for bioH_2_ production.[^94^, ^95^, ^96^] Another crucial parameter in DF bioH_2_ production is the hydraulic retention time (HRT). Short HRTs are employed in continuous stirred‐tank reactor (CSTR) systems to favour acid‐producing bacteria over methanogens.^[97]^ Additionally, a short HRT can facilitate a lower pH level during anaerobic processes.^[98]^ In the context of bioH_2_ generation, a short HRT of 3 days is sufficient for a CSTR system.^[99]^ Furthermore, the cultivation of dark fermentative bacteria can be achieved using mixed cultures or pure cultures. Mixed cultures are preferred for large‐scale applications as they do not require sterile conditions for growth, making them more feasible and cost‐effective on an industrial scale.^[35]^ Notably, the agri‐residues mentioned in this study are lignocellulosic and are favourable for the production of bioH_2,_ as mentioned by Kumar et al.^[100]^

The utilisation of lignocellulosic materials for DF bioH_2_ production holds significant promise for future renewable energy applications. However, a primary challenge hindering global development in this area is the issue of low H_2_ production. Various strategies have been explored to pretreat the inoculum, enhancing H_2_ producers while suppressing H_2_ consumers to boost bioH_2_ production. Heat and acid/alkali/enzymatic pretreatment methods have received the most attention and have demonstrated success in numerous investigations.[^62^, ^64^, ^66^, ^69^, ^70^, ^71^, ^72^, ^74^, ^76^, ^77^, ^82^, ^83^, ^86^, ^101^] Other technologies, including sonication, steam explosion, fungal pretreatment, ozone pretreatment, and hydrothermal pretreatment, have also shown promise in various research studies.[^65^, ^67^, ^73^, ^80^, ^102^] However, further research is required to determine the optimal pretreatment conditions for different substrates, presenting an ongoing challenge.^[103]^ It is worth mentioning that different pretreatment methods may yield varying amounts of bioH_2_, and researchers and practitioners should carefully consider the most suitable pretreatment approach for optimising bioH_2_ production from specific agricultural residues.

In addition to the aforementioned challenges associated with DF bioH_2_ production, another significant hurdle is the collection, handling, and management of agricultural residues across different states/regions in India. This process is complex and can be costly. The primary objective of using these residues as feedstocks for bioH_2_ generation through dark fermentation is to assess whether it can offer economic and environmental benefits in addressing India's fossil fuel challenges. To achieve this, a well‐structured agricultural residue management system is imperative in every state/region to effectively utilise residues for biofuel production, such as DF bioH_2_. Ensuring a consistent and adequate supply of agricultural residues throughout the year is essential for the successful operation of bioH_2_ production facilities.

This study underscores that due to geographical and agricultural constraints, the same type of feedstock cannot be universally applied across all states in India for bioH_2_ production. Nonetheless, decentralised DF bioH_2_ production initiatives can be implemented in various regions with locally abundant high‐yield residues. Considering the estimated bioH_2_ production results from 7 crop residues, the top states favourable for decentralised bioH_2_ production pant implementation are‐ West Bengal, Uttar Pradesh, Karnataka, Punjab, Madhya Pradesh, Maharashtra, and Bihar. While many researchers have explored the prospects of bioH_2_ production from different residues available in India, there remains a gap in research on large‐scale implementation to address the energy crisis. Future research endeavours involving various regional crop residues and diverse process combinations are expected to yield further insights into this field. Furthermore, conducting techno‐economic and environmental analyses of DF bioH_2_ production from different agricultural residues will empower India's bioenergy sector by encouraging increased investment in renewable bioH_2_ production.

## 4 Conclusions

This study estimates India's bioH_2_ production potential through dark fermentation from 7 major crop residues. Based on the crop statistics of the 2018–19 fiscal year, the estimated potential amount and associated bioenergy of these major crop residues in different states/regions of India has also been studied. Out of the total estimated gross residue of 565.889 MT from these seven crops, only 29.74 % (168.324 MT) was identified as surplus residue. Notably, the top three surplus crop residue‐producing states in 2018–19 were West Bengal (for rice straw and rice husk), Uttar Pradesh (for wheat straw, sugarcane tops and sugarcane bagasse) and Karnataka (for maize stalk and maize cob).

The minimum (without residue pretreatment) and maximum (with residue pretreatment) estimated potential of DF bioH_2_ production in India from the 7 residues considered in this study are 8081.293 MCUM and 23537.402 MCUM, respectively. This is equivalent to a bioH_2_ energy potential spanning 103.018 to 300.048 PJ per year, which is 0.27–0.77 % of India's primary energy consumption in 2019. We have briefly discussed the challenges associated with establishing bioH_2_ production plants in India, emphasising the positive impact of crop residue pretreatment on bioH_2_ yield. An overall increase of 191 % in estimated bioH_2_ production was seen from the dark fermentation of pretreated residues compared to that of raw residues.

This study has certain limitations in data collection, including RPR, SRF, and bioH_2_ yield data for diverse agricultural wastes. In reality, these statistics may vary by location and season. In some instances, specific assumptions were made to generate study outcomes, as noted in relevant sections. However, for most data‐gathering operations, standard accessible literature was consulted. More recent data on RPR, SRF and localised bioH_2_ yield would allow for a more precise assessment of the crop residue‐based bioH_2_ and associated energy production potential. Practical implementation should focus on integrating bioH_2_ production into existing agricultural waste management systems in India. For instance, decentralised biohydrogen plants can be established near agricultural hubs, leveraging surplus residues as feedstock. This approach not only enhances scalability and feasibility but also reduces transportation costs and emissions associated with residue management.

The findings of this study are intended to provide a deeper understanding of the availability of bioH_2_ through DF in India, aiming to benefit the implementation of major regional crop residue‐based renewable energy generation and related legislative efforts. This research is also poised to support future planning and research in decentralised hydrogen energy production projects using agricultural residue biomass across various regions of India, which can contribute significantly to the socio‐economic growth and advancement of India's renewable energy sector.

Future research should prioritise understanding and optimising specific pretreatment mechanisms, such as acidic, alkaline, and enzymatic processes, to enhance BioH_2_ yields. This is critical within the broader scope of DF biohydrogen production, as pretreatment significantly influences the breakdown of complex biomass structures. Additionally, examining and optimising the influence of feedstock chemical composition on bioH_2_ production efficiency is vital for maximising yields and improving overall process efficiency. Detailed, region‐specific, and feedstock‐specific studies could provide valuable insights for tailoring pretreatment approaches to the unique characteristics of various biomass types. Building on these findings, future work involving experimental validation and modelling of pretreatment methods across a broader spectrum of crop residues in India would significantly expand the knowledge base for bioH_2_ production, supporting the effective implementation of biohydrogen as a viable and sustainable solution to the energy crisis. This data‐driven research framework can be readily implemented in other regions worldwide for DF bioH_2_ production from agricultural residues.

## 5 Abbreviations

EJ: Exajoule
HE: BioH_2_ energy
PJ: Petajoule
UT: Union territories
MJ: Megajoule
COP: Conference of the Parties
kWh: Kilowatt‐hour
GHG: Greenhouse gas
MT: Million tonnes
MeOH: Methanol
HV: Heating value
GHG: Greenhouse gas emissions
BioH_2_
: Biohydrogen
DF: Dark fermentation
Y_R_
: BioH_2_ yield from the raw substrate
Y_P_
: BioH_2_ yield from the pretreated substrate
RPR: Residue production ratio
MCUM: Million cubic meters
SRF: Surplus residue factor
DF bioH_2_
: Biohydrogen produced via dark fermentation
GR: Gross residue
SR: Surplus residue
HY: BioH_2_ yield
HP: BioH_2_ production

## 6 Consent for Publication

All authors agree to publish this article.

## 7 Ethics Approval and Consent to Participate

Not applicable.

## 8 Funding

Dr. Ahmed I. Osman and Prof. David W. Rooney wish to acknowledge the support of The Bryden Centre project (Project ID VA5048), which was awarded by The European Union's INTERREG VA Programme, managed by the Special EU Programmes Body (SEUPB), with match funding provided by the Department for the Economy in Northern Ireland and the Department of Business, Enterprise and Innovation in the Republic of Ireland.

## 9 Disclaimer

The views and opinions expressed in this systematic review do not necessarily reflect those of the European Commission or the Special EU Programmes Body (SEUPB).

## Author Contributions

Tanmay J. Deka: Writing – original draft, Writing – review & editing, Software, Conceptualisation, Investigation, Methodology, Data analysis, Visualisation.

Ahmed I. Osman: Conceptualisation, Writing – review & editing, Methodology, Investigation, Proofreading, Supervision, Funding acquisition.

Mohamed Farghali: Writing‐ review & editing, Proofreading.

Ahmed Alengebawy: Writing‐ review & editing, Literature analysis and Introduction.

Debendra C. Baruah: Writing – review & editing, Formal analysis, Validation, Proofreading.

David W. Rooney: Writing – review & editing, Software, Project administration, Supervision, Funding acquisition.

All authors read and approved the final manuscript.

## Conflict of Interests

The authors declare no conflict of interest.

10

## Supporting information

As a service to our authors and readers, this journal provides supporting information supplied by the authors. Such materials are peer reviewed and may be re‐organized for online delivery, but are not copy‐edited or typeset. Technical support issues arising from supporting information (other than missing files) should be addressed to the authors.

Supporting Information

## Data Availability

All data are fully available without restriction.
