# Supplementary material for: Quantitative Modelling of Biohydrogen Production from Indian Agricultural Residues via Dark Fermentation
Source: ChemistryOpen. 2025 Mar 29;14(5):e202400095. doi: 10.1002/open.202400095 (PMC12075106; doi:10.1002/open.202400095)
Supplement: Supplementary file 1 — Supporting Information [file OPEN-14-e202400095-s001.pdf]

ChemistryOpen

Supporting Information

Quantitative Modelling of Biohydrogen Production from Indian Agricultural Residues via Dark Fermentation

Tanmay J. Deka, Ahmed I. Osman,* Mohamed Farghali, Ahmed Alengebawy, Debendra C. Baruah, and David W. Rooney

Supplementary Tables

Quantitative Modelling of Biohydrogen Production from Indian Agricultural Residues via Dark Fermentation

Tanmay J. Deka[1], Ahmed I. Osman [1]*, Mohamed Farghali[2,3], Ahmed Alengebawy[4], Debendra C. Baruah[5] and David W. Rooney[1]

[1] School of Chemistry and Chemical Engineering, Queen's University Belfast, Belfast, United Kingdom

[2] Department of Agricultural Engineering and Socio-Economics, Kobe University, Kobe 657-8501, Japan.

[3] Department of Animal and Poultry Hygiene & Environmental Sanitation, Faculty of Veterinary Medicine, Assiut University, Assiut 71526, Egypt.

[4] College of Engineering, Huazhong Agricultural University, 430070, Wuhan, China.

[5] Department of Energy, Tezpur University, 784001, Tezpur, India

*Corresponding Authors:

Dr Ahmed Osman; Email: aosmanahmed01@qub.ac.uk;

Fax: +44 2890 97 4687

Tel.: +44 2890 97 4412

Address: School of Chemistry and Chemical Engineering, Queen's University Belfast, David Keir Building, Stranmillis Road, Belfast BT9 5AG, Northern Ireland, United Kingdom

Table S1: Supplementary data for Rice crop production, gross & surplus residue estimation, bioH_2 production & bioH_2 energy potential													
	Rice Crop data 2018-19			Rice Straw residue (in Million tonnes)		Rice Straw residue (in Million tonnes)		BioH_2 production potential (in MCUM)			BioH_2 energy potential (in PJ)		
Major States	Area (in Million Ha)	Production (in Million tonnes)	Yield (in Kg/Ha)	Gross Residue	Surplus Residue	Gross Residue	Surplus Residue	Raw Rice Straw	Pretreated Rice Straw	Pretreated Rice Husk	Raw Rice Straw	Pretreated Rice Straw	Pretreated Rice Husk
				RPR= 1.5	SRF= 0.24	RPR= 0.2	SRF= 0.71	Y_R= 50.85 mL/g dry residue	Y_P = 102.3 mL/g dry residue	Yp= 473.1 mL/g dry residue	CV= 0.0127478 PJ/MCUM		
West Bengal	5.52	16.05	2906	24.075	5.778	3.210	2.279	293.811	591.089	1078.242	3.745	7.535	13.745
Uttar Pradesh	5.75	15.54	2704	23.310	5.594	3.108	2.207	284.475	572.307	1043.980	3.626	7.296	13.309
Punjab	3.1	12.82	4132	19.230	4.615	2.564	1.820	234.683	472.135	861.250	2.992	6.019	10.979
Andhra Pradesh	2.21	8.25	3733	12.375	2.970	1.650	1.172	151.025	303.831	554.237	1.925	3.873	7.065
Odisha	3.71	7.31	1972	10.965	2.632	1.462	1.038	133.817	269.213	491.087	1.706	3.432	6.260
Telangana	1.95	6.7	3436	10.050	2.412	1.340	0.951	122.650	246.748	450.107	1.564	3.146	5.738
Chhattisgarh	3.61	6.53	1810	9.795	2.351	1.306	0.927	119.538	240.487	438.687	1.524	3.066	5.592
Tamil Nadu	1.72	6.45	3748	9.675	2.322	1.290	0.916	118.074	237.541	433.312	1.505	3.028	5.524
Bihar	3.18	6.04	1902	9.060	2.174	1.208	0.858	110.568	222.441	405.768	1.410	2.836	5.173
Assam	2.46	5.14	2086	7.710	1.850	1.028	0.730	94.093	189.296	345.306	1.200	2.413	4.402
Haryana	1.45	4.52	3121	6.780	1.627	0.904	0.642	82.743	166.463	303.655	1.055	2.122	3.871
Madhya Pradesh	1.98	4.5	2270	6.750	1.620	0.900	0.639	82.377	165.726	302.311	1.050	2.113	3.854
Others	7.16	16.58	2316	24.870	5.969	3.316	2.354	303.514	610.608	1113.848	3.869	7.784	14.199
All India	43.79	116.42	2659	174.630	41.911	23.284	16.532	2131.185	4287.516	7821.119	27.168	54.656	99.702

Table S2: Supplementary data for Wheat crop production, gross & surplus residue estimation, bioH_2 production & bioH_2 energy potential									
	Wheat Crop data 2018-19			Wheat Straw residue (in Million tonnes)		BioH_2 production potential (in MCUM)		Wheat straw BioH_2 energy potential (in PJ)	
**Major States**	**Area (in Million Ha)**	**Production (in Million tonnes)**	**Yield (in Kg/Ha)**	**Gross Residue**	**Surplus Residue**	**Raw Wheat straw**	**Pretreated Wheat straw**	**Raw Wheat Straw**	**Pretreated Wheat Straw**
				RPR= 1.5	SRF= 0.17	Y_R=32.76 mL/g dry residue	Y_P = 84.41 mL/g dry residue	CV= 0.0127478 PJ/MCUM	
Uttar Pradesh	9.54	32.75	3432	49.125	8.351	273.587	704.929	3.488	8.986
Punjab	3.52	18.24	5183	27.360	4.651	152.373	392.608	1.942	5.005
Madhya Pradesh	5.52	15.47	2802	23.205	3.945	129.233	332.985	1.647	4.245
Haryana	2.55	12.57	4925	18.855	3.205	105.007	270.564	1.339	3.449
Rajasthan	3	10.49	3501	15.735	2.675	87.631	225.793	1.117	2.878
Bihar	2.11	6.15	2922	9.225	1.568	51.376	132.376	0.655	1.688
Gujarat	0.8	2.4	3010	3.600	0.612	20.049	51.659	0.256	0.659
Maharashtra	0.57	0.95	1666	1.425	0.242	7.936	20.448	0.101	0.261
Uttarakhand	0.33	0.94	2880	1.410	0.240	7.853	20.233	0.100	0.258
Himachal Pradesh	0.32	0.57	1774	0.855	0.145	4.762	12.269	0.061	0.156
Others	0.89	1.66	1867	2.490	0.423	13.867	35.731	0.177	0.456
All India	29.14	102.19	3507	153.285	26.059	853.675	2199.594	10.883	28.040

Table S3: Supplementary data for Maize crop production, gross & surplus residue estimation, bioH_2 production & bioH_2 energy potential

	Maize Crop data 2018-19			Maize Stalk residue (in Million tonnes)		Maize Cob residue (in Million tonnes)		BioH_2 production potential (in MCUM)				BioH_2 energy potential (in PJ)			
Major States	Area (in Million Ha)	Production (in Million tonnes)	Yield (in Kg/Ha)	Gross Residue	Surplus Residue	Gross Residue	Surplus Residue	Raw Maize Stalk	Pretreated Maize Stalk	Raw Maize Cob	Pretreated Maize Cob	Raw Maize Stalk	Pretreated Maize Stalk	Raw Maize Cob	Pretreated Maize Cob
				RPR= 2	SRF= 0.2	RPR= 0.3	SRF= 0.38	Y_R=92.9 mL/g dry residue	Y_P = 163.1 mL/g dry residue	Y_R=93.1 mL/g dry residue	Yp=120.5 mL/g dry residue	CV= 0.0127478 PJ/MCUM			
Karnataka	1.34	3.73	2777	7.460	1.492	1.119	0.425	138.607	243.345	39.588	51.239	1.767	3.102	0.505	0.653
Madhya Pradesh	1.37	3.68	2697	7.360	1.472	1.104	0.420	136.749	240.083	39.057	50.552	1.743	3.061	0.498	0.644
Bihar	0.68	3.02	4451	6.040	1.208	0.906	0.344	112.223	197.025	32.052	41.486	1.431	2.512	0.409	0.529
Tamil Nadu	0.38	2.51	6551	5.020	1.004	0.753	0.286	93.272	163.752	26.640	34.480	1.189	2.087	0.340	0.440
Telangana	0.56	2.03	3658	4.060	0.812	0.609	0.231	75.435	132.437	21.545	27.886	0.962	1.688	0.275	0.355
Rajasthan	0.86	1.96	2285	3.920	0.784	0.588	0.223	72.834	127.870	20.802	26.925	0.928	1.630	0.265	0.343
Maharashtra	0.95	1.93	2032	3.860	0.772	0.579	0.220	71.719	125.913	20.484	26.512	0.914	1.605	0.261	0.338
Andhra	0.27	1.56	5861	3.120	0.624	0.468	0.178	57.970	101.774	16.557	21.430	0.739	1.297	0.211	0.273
Uttar Pradesh	0.73	1.53	2090	3.060	0.612	0.459	0.174	56.855	99.817	16.239	21.018	0.725	1.272	0.207	0.268
West Bengal	0.24	1.38	5780	2.760	0.552	0.414	0.157	51.281	90.031	14.646	18.957	0.654	1.148	0.187	0.242
Others	1.81	3.89	2149	7.780	1.556	1.167	0.443	144.552	253.784	41.286	53.437	1.843	3.235	0.526	0.681
All India	9.18	27.23	2965	54.460	10.892	8.169	3.104	1011.867	1776.485	289.003	374.059	12.899	22.646	3.684	4.768

Table S4: Supplementary data for Sugarcane crop production, gross & surplus residue estimation, bioH_2 production & bioH_2 energy potential															
	Sugarcane Crop data 2018-19			Sugarcane tops (ST) residue (in Million tonnes)		Sugarcane bagasse (SB) residue (in Million tonnes)		BioH_2 production potential (in MCUM)				BioH_2 energy potential (in PJ)			
**Major States**	**Area (in Million Ha)**	**Production (in Million tonnes)**	**Yield (in Kg/Ha)**	**Gross Residue**	**Surplus Residue**	**Gross Residue**	**Surplus Residue**	**Raw ST**	**Pretreated ST**	**Raw SB**	**Pretreated SB**	**Raw ST**	**Pretreated ST**	**Raw SB**	**Pretreated SB**
				RPR= 0.05	SRF= 0.19	RPR= 0.33	SRF= 0.50	Y_R=43.17 mL/g dry residue	Y_P = 60.6 mL/g dry residue	Y_R=55 mL/g dry residue	Yp=103.72 mL/g dry residue	CV= 0.0127478 PJ/MCUM			
Uttar Pradesh	2.22	179.71	80807	8.986	1.707	59.304	29.652	73.702	103.459	1630.868	3075.521	0.940	1.319	20.790	39.206
Maharashtra	1.16	92.44	79500	4.622	0.878	30.505	15.253	37.911	53.218	838.893	1582.000	0.483	0.678	10.694	20.167
Karnataka	0.51	42.01	83000	2.101	0.399	13.863	6.932	17.229	24.185	381.241	718.951	0.220	0.308	4.860	9.165
Tamil Nadu	0.16	16.21	98240	0.811	0.154	5.349	2.675	6.648	9.332	147.106	277.415	0.085	0.119	1.875	3.536
Gujarat	0.17	12.04	71971	0.602	0.114	3.973	1.987	4.938	6.931	109.263	206.050	0.063	0.088	1.393	2.627
Bihar	0.23	11.66	51695	0.583	0.111	3.848	1.924	4.782	6.713	105.815	199.547	0.061	0.086	1.349	2.544
Andhra Pradesh	0.1	8.09	79325	0.405	0.077	2.670	1.335	3.318	4.657	73.417	138.451	0.042	0.059	0.936	1.765
Punjab	0.1	7.77	81828	0.389	0.074	2.564	1.282	3.187	4.473	70.513	132.974	0.041	0.057	0.899	1.695
Haryana	0.09	7.57	80367	0.379	0.072	2.498	1.249	3.105	4.358	68.698	129.551	0.040	0.056	0.876	1.651
Madhya Pradesh	0.12	6.96	58950	0.348	0.066	2.297	1.148	2.854	4.007	63.162	119.112	0.036	0.051	0.805	1.518
Uttarakhand	0.09	6.33	69553	0.317	0.060	2.089	1.044	2.596	3.644	57.445	108.330	0.033	0.046	0.732	1.381

Telangana	0.04	2.8	70000	0.140	0.027	0.924	0.462	1.148	1.612	25.410	47.919	0.015	0.021	0.324	0.611
Others	0.12	6.57	53358	0.329	0.062	2.168	1.084	2.694	3.782	59.623	112.438	0.034	0.048	0.760	1.433
All India	5.11	400.16	78248	20.008	3.802	132.053	66.026	164.112	230.372	3631.45<br>2	6848.258	2.092	2.937	46.293	87.300

Table S5: Supplementary data related to the gross & surplus residue estimation, bioenergy potential for all considered agri-residues			
**Agri-residue biomass**	**Gross residue (in MT)**	**Surplus residue (in MT)**	**Surplus bioenergy (in PJ) ***
Rice straw	174.63	41.911	603.521
Rice husk	23.284	16.532	255.414
Wheat straw	153.285	26.058	449.508
Maize stalk	54.46	10.892	186.035
Maize cob	8.169	3.104	56.993
Sugarcane tops	20.008	3.801	55.996
Sugarcane bagasse	132.053	66.026	1158.103
Total	565.889	66.026	2765.57

*Same HV for raw and pretreated assumed
